# Impact of reduced anthropogenic emissions and century flood on the phosphorus stock, concentrations and loads in the Upper Danube

**DOI:** 10.1016/j.scitotenv.2015.02.087

**Published:** 2015-06-15

**Authors:** Ottavia Zoboli, Alberto Viglione, Helmut Rechberger, Matthias Zessner

**Affiliations:** a Centre for Water Resource Systems, Vienna University of Technology, Karlsplatz 13/222, 1040 Vienna, Austria; b Institute for Water Quality, Resource and Waste Management, Vienna University of Technology, Karlsplatz 13/226, 1040 Vienna, Austria; c Institute of Hydraulic Engineering and Water Resources Management, Vienna University of Technology, Karlsplatz 13/222, 1040 Vienna, Austria

**Keywords:** Danube, Phosphorus, Flood, Stock, Mobilization

## Abstract

Patterns of changes in the concentration of total and soluble reactive phosphorus (TP, SRP) and suspended sediments at different flow levels from 1991 to 2013 in the Austrian Danube are statistically analyzed and related to point and diffuse emissions, as well as to extreme hydrological events. Annual loads are calculated with three methods and their development in time is examined taking into consideration total emissions and hydrological conditions. The reduction of point discharges achieved during the 1990s was well translated into decreasing TP and SRP baseflow concentrations during the same period, but it did not induce any change in the concentrations at higher flow levels nor in the annual transport of TP loads. A sharp and long-lasting decline in TP concentration, affecting all flow levels, took place after a major flood in 2002. It was still visible during another major flood in 2013, which recorded lower TP concentrations than its predecessor. Such decline could not be linked to changes in point or diffuse emissions. This suggests that, as a result of the flood, the river system experienced a significant depletion of its in-stream phosphorus stock and a reduced mobilization of TP rich sediments afterwards. This hypothesis is corroborated by the decoupling of peak phosphorus loads from peak maximum discharges after 2002. These results are highly relevant for the design of monitoring schemes and for the correct interpretation of water quality data in terms of assessing the performance of environmental management measures.

## Highlights

• Decline of baseflow P concentration in the 1990s due to point discharge reduction.
• Despite reduced emissions, high loads in the 1990s due to pool of P rich sediments.
• Sharp and enduring decline of TP concentration at all flow levels after 2002 flood.
• Depletion of P rich sediments and long-lasting low riverine loads after 2002 flood.
• Effects of point discharge reduction visible only after depletion of P rich pool.

## Graphical abstract

**Figure:**
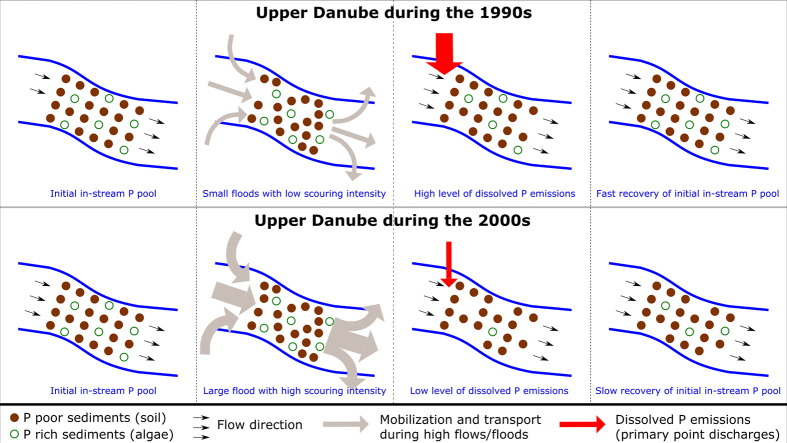

## 1 Introduction

The Danube is the second largest river in Europe and is responsible for almost 60% of the freshwater and for the majority of sediments and nutrients entering the Black Sea (Maksimovic and Makropoulos, 2002; Schreiber et al., 2005). Consequently its elevated transport of phosphorus was identified as one of the main causes of the severe eutrophication that affected the sea during the 1980s and early 1990s (Kroiss et al., 2006). The control of phosphorus pollution in the Danube is therefore of primary importance, both to sustain the ecological health of the river itself, and to reduce the loads transported downstream.

Austria accounts for 10% of the total area of the Danube Basin, which drains more than 96% of its territory (ICPDR, 2014a). In the last 30 years the country has undertaken several efforts to reduce phosphorus emissions. In the 1980s the use of phosphates in detergents was dramatically reduced (Behrendt et al., 2005) and in the 1990s the Austrian edict BGBl. Nr. 180/1991, later replaced by the BGBl. Nr. 210/1996, introduced the mandatory removal of phosphorus in wastewater treatment plants (WWTP). Moreover, the agri-environmental program ӦPUL was launched in the year 2000 to address diffuse nutrient losses (BMLFUW, 2000). In addition to anthropogenic changes, the Upper Danube Basin was also exposed to extreme hydrological conditions. After a succession of flood-poor decades, with the exception of a minor event in 1991, the Austrian Danube was hit first in August 2002 by a flood that, due to its extension and duration, was termed a “century flood”, and then in June 2013 by one of the largest floods to have taken place in the last two centuries (Blöschl et al., 2013). In 2003, on the contrary, the whole Basin experienced a pronounced drought, with below-average rainfalls and above-average temperatures. The relative precipitation recorded in Austria in that year, for example, corresponded to 74% of the long-term annual average (ICPDR, 2014c).

At the beginning of the 1990s an extensive monitoring network was set up by two independent agencies, the Austrian Federal Ministry of Agriculture, Forestry, Environment and Water Management (BMLFUW) and the International Commission for the Protection of the Danube River (ICPDR). It is thus feasible to examine in detail this period of combined anthropogenic changes and extreme hydrological regimes, which represent an exceptional opportunity for exploring interweaving causalities in a large river.

The link between improved wastewater phosphorus removal and decline in phosphorus concentration in European rivers has been demonstrated and quantified in previous studies (Neal et al., 2010; Räike et al., 2003). Conversely, investigations dealing with episodic flood events, total emissions, and their relationship have focused on riverine loads (mass time^− 1^), rather than on concentrations (mass volume^− 1^). Behrendt and Opitz (2000) observed, across 100 basins in Europe, a considerable discrepancy between total emissions and measured transport loads, with the latter being significantly lower. They found that the difference increased as a function of the specific runoff of the basins. Similar findings were reported by Zessner and Kroiss (1999) for the Upper Danube. The reason for such discrepancies lies in the retention process, which has been increasingly recognized as a relevant mechanism to be further investigated and included in river basin models (de Klein and Koelmans, 2011; Venohr et al., 2011). The retention of phosphorus takes place essentially through deposition and algae growth. The stock generated is then exposed to remobilization during flood events, when peaks of phosphorus are transported downstream and exported to river banks and flooded areas (Zessner et al., 2005; House et al., 1997; Dorioz and Ferhi, 1994; Johnson et al., 1976). According to Stamm et al. (2014), this depletion is replaced by the deposition of particulate phosphorus during the recessional part of the storms, which represents a renewed internal source of baseflow phosphorus. Although this might hold true for small episodic events, the impact of a major flood could be more intense and exert a more profound impact on both the in-stream stock and the concentration, with consequences not only for the peak transport of phosphorus to downstream standing water bodies and to the lateral river system, but also for the river ecology itself.

In this study, time series of water quality data of the Upper Danube are examined to identify patterns of change in phosphorus concentration, and to link them to anthropogenically driven changes and to extreme hydrological conditions. The aim of the work is to assess the performance and effectiveness of environmental management strategies, and to investigate the short-term and long-term impact of large episodic events on the in-stream phosphorus concentration and on the stock of the river system.

To gain a deeper insight into the drivers of shifts in concentration, not only is total phosphorus (TP) analyzed, but also soluble reactive phosphorus (SRP, equivalent to orthophosphate) and suspended sediments (SS). SRP contributes to identifying the impact of point emissions, because it is typically the prevalent phosphorus species in WWTP effluents (Jarvie et al., 2006). SS provides further information regarding diffuse pathways, because particulate-bound phosphorus is the predominant species transported by storm-dependent agricultural runoff and erosion processes (Withers and Jarvie, 2008).

## 2 Materials and methods

### 2.1 Data sets

**Fig. 1:**
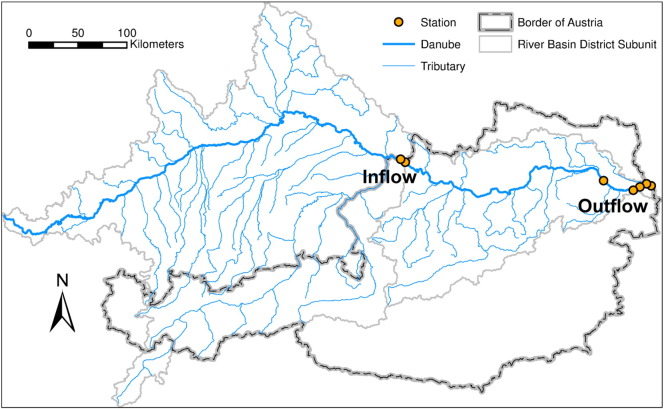
Map of the Upper Danube Basin and of the monitoring stations located at the Austrian Inflow and Outflow.

The Danube was analyzed at its entrance into Austrian territory from Germany (Inflow) and at its exit from Austria (Outflow) (Fig. 1). This enables the phosphorus contribution within the Austrian catchment to be determined.

The study used a collection of different data sources, namely the H_2_O database created and maintained since 1991 by BMLFUW (2014b), and two ICPDR databases, one obtained through a first campaign performed from 1992 to 1998 (Bucharest Declaration data set) and the other through the Transnational Monitoring Network (TNMN) launched in 1996 (ICPDR, 2014b). In addition, for the Outflow a specific sample collected during the flood of August 2002 (Zessner et al., 2005) and a data set of semi-continuous measurements for the entire year 2013 (BMLFUW, 2013) were available (Tables 1 and 2). Since 1991 the monitoring of surface and groundwater water quality in Austria has been regulated by federal legislation (BGBL Nr. 338/1991 replaced by BGBl. II Nr. 479/2006) that specifies the standard analytical procedures to be followed for each parameter. With respect to total phosphorus and phosphorus compounds, at the beginning of the 1990s analyses were required to comply with the Standard Ö-NORM M 6237:1986, which was then replaced by ISO 6878:1998 and later revised by ISO 6878:2004. These standards all maintained the spectrometric determination using ammonium molybdate as basis. Although accredited laboratories are entitled to apply different methods, they are required to prove their equivalence to the standard procedures. These protocols were applied by all data sources considered here. This ensures a substantial consistency of the analytical methods throughout the studied time period, although some minor variations cannot be entirely excluded. In addition, the analyses of each sample have been run in triplicate in accordance with the aforementioned protocols, with the exception of the data set of Zessner et al. (2005), which relies on single tests.

### 2.2 Data set subdivision in time periods and flow intervals

Phosphorus concentration and species fractionation can vary largely as a function of flow levels due to differing primary pathways and driving natural processes. TP concentration generally rises at increasing flow levels due to the higher transport of particulate-bound phosphorus in more turbulent conditions (Zessner and Kroiss, 1999). On the contrary, SRP concentration is expected to be higher at baseflow conditions, because its primary pathway is the quasi-constant WWTP effluent discharge, which becomes more heavily diluted at higher flows (Jarvie et al., 1997). To preserve the information contained in these differences, the flow levels were subdivided into intervals, following the flow duration curve scheme suggested by EPA (2007). The flows were categorized according to the percentage of exceedance, as follows: *High flows* (0–10%), *Moist conditions* (10–40%), *Mid-range flows* (40–60%), *Dry conditions* (60–90%), and *Low flows* (90–100%). The availability of data during the two flood events in 2002 and 2013 at the Outflow also enables the investigation of the differences and changes in time in high flow conditions, which are especially important for the transport of phosphorus. Thus, the interval *High flows* for the Outflow was further subdivided into three ranges of equal length: *High flows*, *Very high flows*, and *Extremely high flows*, 10,570 m^3^ s^− 1^ being the maximum daily discharge measured within the time series. The calculation of the cumulative frequency was based on two data sets of daily mean discharges measured at the same locations, covering the periods 1985–2011 and 1977–2013 for the Inflow and Outflow, respectively (BMLFUW, 2014a). The flow intervals obtained following this procedure are shown in Table 3.

Studies have shown that low frequency sampling can lead to large errors in the calculation of riverine phosphorus loads (Johnes, 2007; Cassidy and Jordan, 2011). Similar findings were presented by Skarbøvik et al. (2012) with regard to estimating the mean concentration of suspended sediments, which could also be extended to particulate contaminants like phosphorus. Thus, the fact that the available data sets mostly rely on monthly or bi-weekly measurements impedes a reliable analysis of rapid inter-annual variations. This study focuses instead on longer-term changes, for which purpose the time series was subdivided into periods, based on the rationales described in Table 4.

### 2.3 Consistency and combination of the data sets

The data sets were statistically compared, firstly to verify the consistency among different stations and monitoring agencies, and secondly to determine the appropriateness of their combination. The K-sample Anderson–Darling test (Scholz and Stephens, 1987) was chosen to test whether at each flow interval and time period the different samples can be considered to belong to the same distribution. It was applied to the three flow ranges with highest data availability, namely *Dry conditions*, *Mid-range flows*, and *Moist conditions*, with the assumption that they be representative for the whole flow duration curve. For the Outflow and the period T2, the samples within *High flows* were also tested to include the data set of Zessner et al. (2005). The standard statistical significance levels associated to p values were adopted: significant (p < 0.05), very significant (p < 0.01), and highly significant (p < 0.001).

As shown in Tables 5 and 6, the data sets generally present a good level of consistency (i.e. p values greater than 0.05). Therefore, none of the data sets was dismissed and all were merged to obtain the final samples shown in Tables 7 and 8. This notwithstanding, the analyses of the study were reiterated alternately removing the data sets that presented some discrepancies, in order to assess their actual impact on the results and on the conclusions.

### 2.4 Analysis of change in time

The combined data sets (Tables 7 and 8) were statistically analyzed to examine the behavior in time of the TP, SRP and SS mean concentrations. The null hypothesis H_0_ was that the mean concentrations calculated at every flow range did not change significantly from each period to the following one. It is therefore a step trend hypothesis, which is tested through a *t*-test for the significance of the difference between the means of two independent samples (Hirsch et al., 1991). In view of the diverse sample size and possibly unequal variance, the Welch's *t*-test was selected (Welch, 1947).

### 2.5 Load calculation

The calculation of annual TP loads was carried out with three different methods: *M1 — Linear*, *M2 — ICPDR*, and *M3 — Flow intervals*.

#### 2.5.1 Method *M1* — *Linear*

The first calculation procedure (Eq. (1)) is a widely used method, and was applied by Littlewood and Marsh (2005) and by Johnes (2007) as the reference to test other methodologies.

(1) $$L=\frac{K\sum_{i=1}^{n}\left(C_{i}Q_{i}\right)}{\sum_{i=1}^{n}\left(Q_{i}\right)}\cdot {\bar{Q}}_{r}$$

Annual loads *L* are calculated as the product of sampled instantaneous concentration *C*_*i*_ and discharge *Q*_*i*_ (*n* is the number of samples in a year), divided by the sum of sampled discharges and multiplied by the average annual discharge ${\bar{Q}}_{r}$, *K* being a factor to account for measurement units and the duration of the period.

This method has a considerable shortcoming due to the underlying assumption of a linear relationship between load and discharge. As this is usually depicted by an exponential function, Eq. (1) will tend to deliver overestimations when data collected at high flow conditions or during storm events are included, as demonstrated by Cassidy and Jordan (2011).

#### 2.5.2 Method *M2* — *ICPDR*

Eq. (2) shows the calculation procedure officially selected and applied by ICPDR (2000).

(2) $$\begin{array}{ccc} C_{m}=\frac{\sum_{i=1}^{m}\left(C_{i}Q_{i}\right)}{\sum_{i=1}^{m}\left(Q_{i}\right)} & L_{m}=KC_{m}Q_{m} & L=\sum_{m=1}^{12}L_{m} \end{array}$$

The method is based on the calculation of monthly loads *L*_*m*_, which are obtained as the product of average monthly discharge *Q*_*m*_ and average monthly concentration *C*_*m*_ (*m* is the number of samples per month), *K* being a factor to account for measurement units and the number of days in each month. *C*_*m*_ is the product of the measured concentrations and discharge values, divided by the sum of sampled discharges.

In comparison to *M1 — Linear*, this procedure adds the inclusion of seasonality. Therefore, it indirectly takes into account, to a certain degree, the relationship between TP concentration and discharge.

#### 2.5.3 Method *M3* — *Flow intervals*

A specific calculation procedure was developed on the one hand to appropriately consider the relationship between TP concentration and discharge, and on the other hand to examine the impact that the shifts in concentration exerted on the total riverine transport. As shown in Eq. (3), the annual loads *L* are obtained as the sum of daily loads, calculated as the product of each daily discharge and the mean concentration of the respective flow interval (*Q*_*l*_, *Q*_*d*_, *Q*_*m*_, *Q*_*mo*_, *Q*_*h*_, *Q*_*v*_, *Q*_*e*_ being daily discharge values and *μ*_*l*_, *μ*_*d*_, *μ*_*m*_, *μ*_*mo*_, *μ*_*h*_, *μ*_*v*_, *μ*_*e*_ mean concentrations at *Low flows*, *Dry conditions*, *Mid-range flows*, *Moist conditions*, *High flows*, *Very high flows*, and *Extremely high flows*, respectively), where *K* is a factor to account for measurement units. For each year, the mean concentrations of the corresponding time period are applied.

(3) $$\begin{array}{l} L=K(\sum_{l,d,m,mo,h,v,e=0}^{n}\left(Q_{l}\mu_{l}\right)+\left(Q_{d}\mu_{d}\right)+\left(Q_{m}\mu_{m}\right)+\left(Q_{mo}\mu_{mo}\right)+\\ \;+Q_{h}\mu_{h})+\left(Q_{v}\mu_{v}\right)+\left(Q_{e}\mu_{e}\right)) \end{array}$$

The results of the three methods are compared to the loads that ICPDR has calculated, based on *M2 — ICPDR* and using exclusively the TNMN data sets (ICPDR, 2000, 2001, 2002, 2003, 2004, 2005, 2006, 2007, 2008, 2009, 2010, 2011).

## 3 Results and discussion

### 3.1 Shifts in phosphorus concentration

#### 3.1.1 Reduction of point emissions during the 1990s

**Fig. 2:**
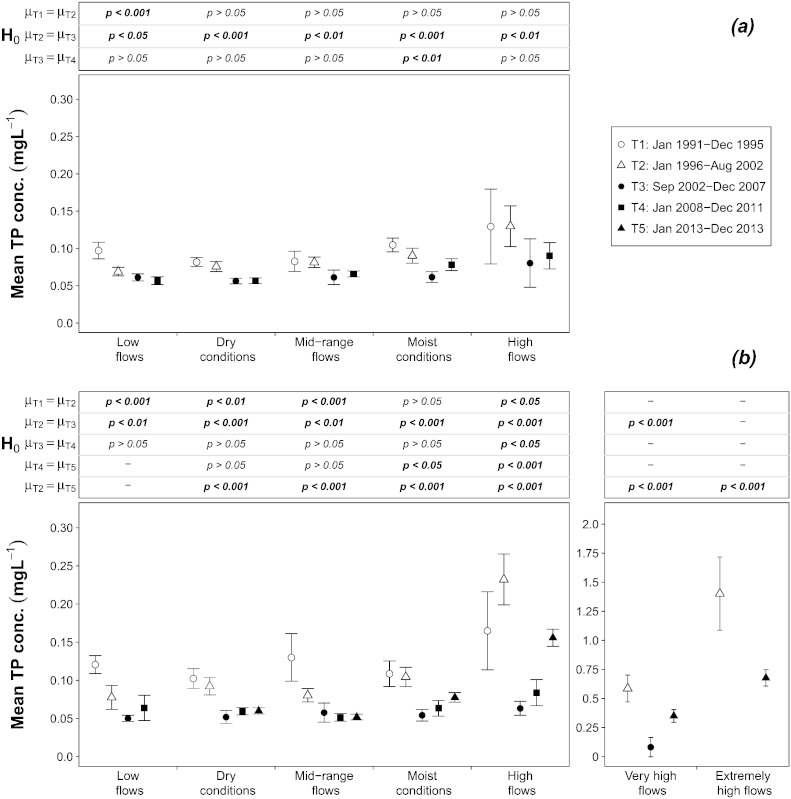
Mean and 95% Confidence Interval of the total phosphorus concentration at each flow interval and time period at: 2a) the Inflow and 2b) the Outflow; significance level of the difference between mean values *μ* among time periods (Welch's *t*-test) are also shown. Statistically significant differences are indicated in bold.

**Fig. 3:**
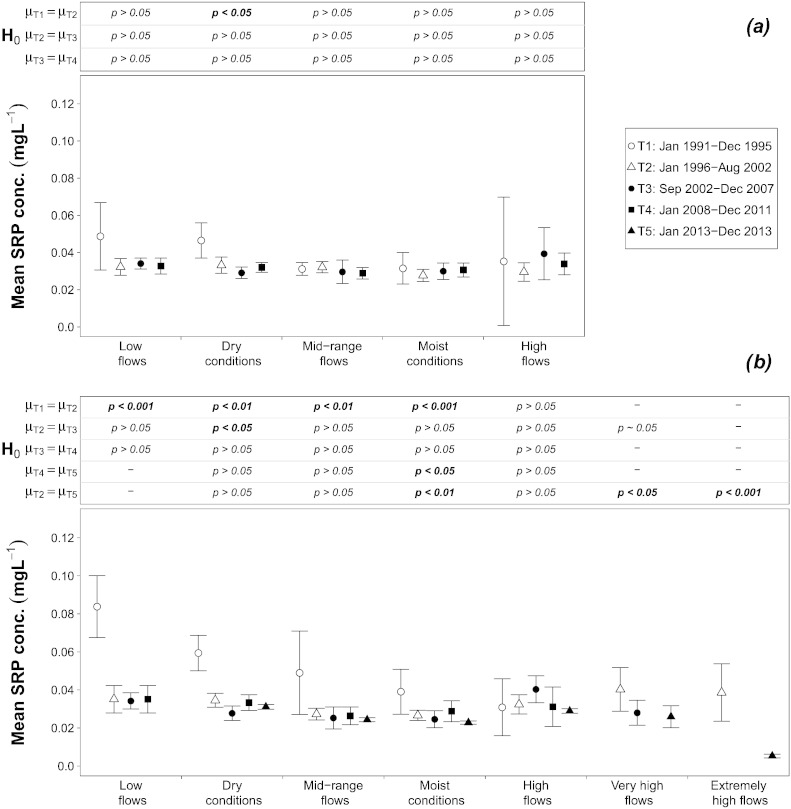
Mean and 95% Confidence Interval of the soluble reactive phosphorus concentration at each flow interval and time period at: 3a) the Inflow and 3b) the Outflow; significance level of the difference between mean values *μ* among time periods (Welch's *t*-test) are also shown. Statistically significant differences are indicated in bold.

The analysis at the Inflow shows that, from the first to the second half of the 1990s, a highly significant decline of the mean TP concentration took place at *Low flows*, from 0.10 to 0.07 mg L^− 1^, whereas at other flow ranges no variation was found (Fig. 2a). At the Outflow the change was more pronounced, with a clear reduction of the TP concentration at *Low flows* (from 0.12 to 0.08 mg L^− 1^), *Dry conditions* (from 0.10 to 0.09 mg L^− 1^), and *Mid-range flows* (from 0.13 to 0.08 mg L^− 1^) (Fig. 2b). The analysis of SRP depicts similar patterns. At the Inflow the calculated mean SRP concentrations at *Low flows* and *Dry conditions* both decreased from 0.05 mg L^− 1^ to 0.03 mg L^− 1^, but this was found to be statistically significant only for the latter, whereas for *Low flows* the samples were too small and displayed too much variability (Fig. 3a). At the Outflow the decline was larger and broader, with a reduction at *Low flows* from 0.09 to 0.04 mg L^− 1^, at *Dry conditions* from 0.06 to 0.03 mg L^− 1^, at *Mid-range flows* from 0.05 to 0.03 mg L^− 1^, and at *Moist conditions* from 0.04 to 0.03 mg L^− 1^ (Fig. 3b).

Such shifts reflect the improvements achieved during this period in the efficiency of phosphorus removal in WWTP. Austrian WWTP gradually increased their average transfer of phosphorus contained in receiving wastewater to sewage sludge from 50% in 1995 to 82% in 2001 (BMLFUW, 1996, 2008). In the first half of the 1990s, German WWTP achieved the target of maximum 1 mg L^− 1^ TP in the effluent, equivalent to an approximately 80% removal rate (TMLNU, 2009), which explains the delayed decrease of TP and SRP concentrations at the Outflow when compared to the Inflow.

The link between the diminished TP concentration and the reduced point emissions is supported by the fact that the decline was detected only at low up to mid-flow conditions, whereas at higher flows it remained constant or even increased, since quasi-constant effluent discharge from WWTP becomes less diluted in drier conditions. This causality is evidenced by the reduction of SRP, a typically dominant phosphorus species in WWTP effluents.

The decline of TP and SRP concentrations in low flow conditions, as a result of the reduced phosphorus load in WWTP effluents, is in accordance with the findings of Neal et al. (2010), which showed a consistent decrease of SRP in the River Thames, induced by the augmented phosphorus removal in WWTP and mostly visible at baseflow conditions. Although the observed trend was similar, the range of variation was very different, since the SRP concentration in the Thames started from 1.6 mg L^− 1^ in 1997 and reached approximately 0.4 mg L^− 1^ in 2006, still 10 times higher than in the Upper Danube. The reasons for these large differences may include the higher population density in the catchment of the River Thames and the still relatively low phosphorus removal rate in the UK (approximately 57% in the year 2009) (Cooper and Carliell-Marquet, 2013).

#### 3.1.2 Consequences of the major flood of August 2002

The analysis shows that, after the major flood of August 2002, a significant and long-lasting decline in the TP concentration occurred. The mean TP concentration at the Inflow varied from 0.07 mg L^− 1^ at *Low flows* to 0.13 mg L^− 1^ at *High flows* before the flood, but ranged from 0.06 mg L^− 1^ to 0.08 mg L^− 1^ afterwards (Fig. 2a). At the Outflow this effect was even more visible. The mean TP concentrations reached very high values of 0.23, 0.54 and 1.25 mg L^− 1^ at *High flows*, *Very high flows* and *Extremely high flows*, respectively. After the flood, the range at all flow levels was reduced to 0.05–0.08 mg L^− 1^ (Fig. 2b). The decline was not only sharp, but also enduring. At both locations the T4 period (2008–2012) was characterized by only slight and inconsistent increases, found to be significant only at *Moist conditions* for the Inflow (Fig. 2a) and at *High flows* for the Outflow (Fig. 2b). Furthermore, the analysis of the semi-continuous data set collected at the Outflow in 2013 shows that the TP concentration persisted at the same low levels at *Dry conditions* and *Mid-range flows* (Fig. 2b). It increased at *Moist conditions* and *High flows*, but never reaching the higher values measured before and during the 2002 flood. The repetition of the analyses alternately excluding the data sets that had presented problems of inconsistencies led to the same results, which means that the few detected discrepancies were offset by the extent of the changes in time. The only effect on the results was a slightly lower statistical significance caused by the reduced size of the samples.

The observed shift in TP concentration cannot be explained through a reduction of point emissions, because in 2002 both Germany and Austria had already achieved a very high removal rate of phosphorus from wastewater – more than 80% – leaving scope for only minor further improvements. This is confirmed by the behavior of the mean SRP concentration, which at both locations remained almost constant in the range of 0.03–0.04 mg L^− 1^ at all flow intervals (Fig. 3a and b).

**Fig. 4:**
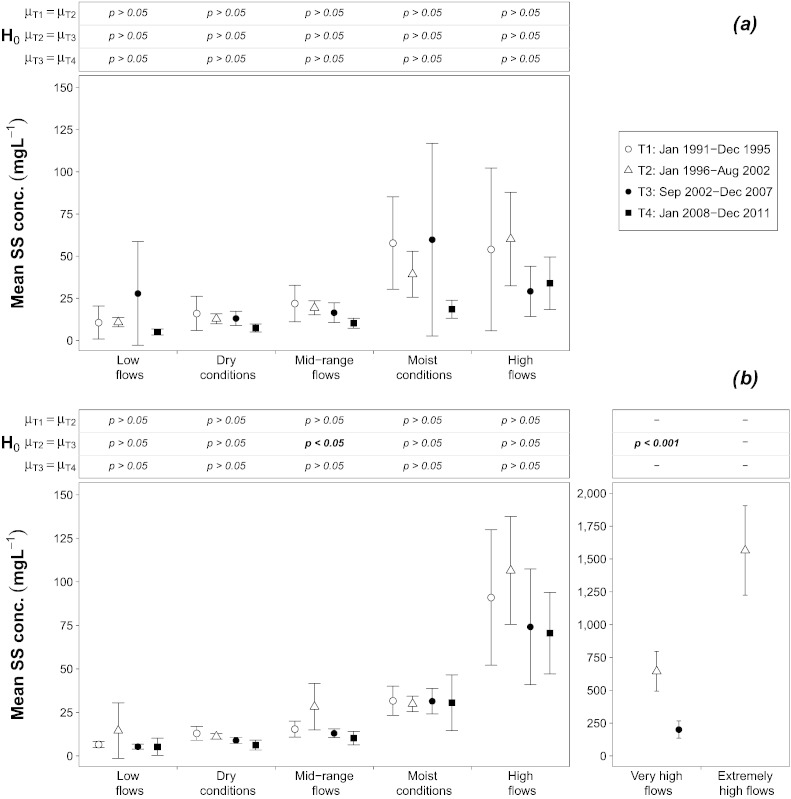
Mean and 95% Confidence Interval of the suspended sediment concentration at each flow interval and time period at: 4a) the Inflow and 4b) the Outflow; significance level of the difference between mean values *μ* among time periods (Welch's *t*-test) are also shown. Statistically significant differences are indicated in bold.

On the other hand, the analysis of the SS (Fig. 4a and b) does not provide any evidence that, following the flood, the Danube experienced a reduced turbidity and transport of sediments, which could have explained the decrease of TP concentration, especially at high flow conditions. Therefore, it was the phosphorus content of the sediments that declined. The very high amount of phosphorus accumulated through decades of fertilization in Austrian agricultural soils (Egle et al., 2014) excludes the possibility that the phosphorus content of eroded sediments decreased significantly. According to Behrendt et al. (2005), diffuse phosphorus emissions in the Danube river system remained relatively constant during the last decades of the 20th century. With respect to the early 2000s, Zessner et al. (2011b) carried out a detailed study aimed at assessing the impact of the agri-environmental program ÖPUL, launched in 2000, on nutrient emissions in Upper Austria. The most important measures implemented were the use of winter cover crops and the strip-till practice, both aimed at preventing soil loss. Given these measures can decrease emissions up to 50%–70% and that they were applied respectively to 20% and 10% of available arable land, it was estimated that their implementation led to a total decrease of approximately 10% of the phosphorus emissions. However, this 10% reduction regards exclusively the emissions associated with agricultural erosion, and this in turn accounts for approximately 40% of total diffuse phosphorus emissions (the rest being mainly split among urban runoff, natural erosion and groundwater pathways). In view of the general implementation status of the ÖPUL program, these results are also applicable to the rest of Austria. Therefore, the contribution of the changes in diffuse emissions to determining such a steep decline of the in-stream TP concentration can be regarded as unimportant.

**Fig. 5:**
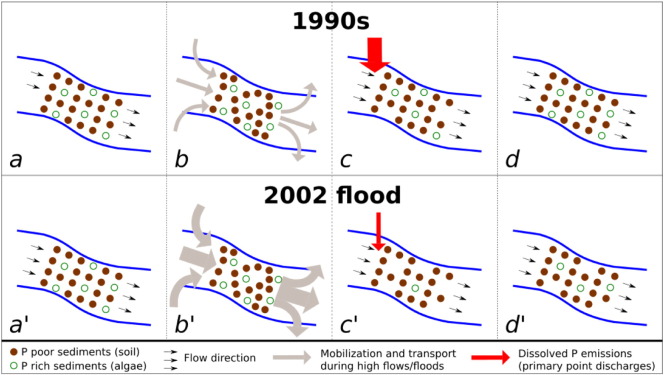
Schematic illustration of the main processes affecting the phosphorus in-stream pool during the observed time series: *a*) initial pool during the 1990s; *b*) transport and mobilization during high flows and minor flood events; *c*) small depletion of phosphorus rich sediments and high levels of dissolved phosphorus emissions; *d*) quick recovery of in-stream pool due to high availability of soluble reactive phosphorus for algae growth; *a′*) initial pool before 2002 flood; *b′*) transport and mobilization during 2002 flood; *c′*) strong depletion of in-stream phosphorus rich sediments and low dissolved phosphorus emissions; *d′*) slow recovery of in-stream pool due to low availability of soluble reactive phosphorus for algae growth.

As a result, the only convincing explanation for the sharp and enduring decline of TP concentration in the Danube after the flood is a reduced mobilization of phosphorus within the river, resulting from a combined effect of the strong event and lower point emissions. It can be hypothesized that the flood intensely scoured the river bed, removing the pool of phosphorus primarily embedded in algae mass. Although algae represent a minor fraction of the total stream sediments, their average phosphorus content is considerably higher than that of soil particles, the first being approximately 1–2% (Borchardt and Azad, 1968; Benedini and Tsakiris, 2013) and the latter ranging between 0.02–1.14% (Koljonen and Darnley, 1994; Noll et al., 2009). This explains the strong reduction of TP concentration without significant changes in the total SS. As a result of point discharge reduction, the lower availability of soluble and easily available phosphorus hindered rapid algae growth, delaying the regeneration of the internal stock, which clarifies the long duration of the TP decline. These mechanisms are presented schematically in Fig. 5.

#### 3.1.3 Comparison of major floods in 2002 and 2013

**Fig. 6:**
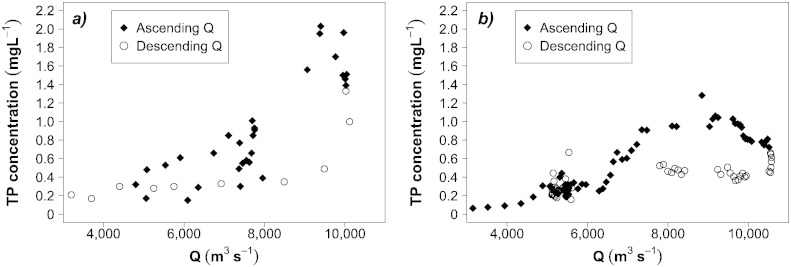
TP concentration measured at the Outflow at ascending and descending discharges during: 5a) August 2002 flood and 5b) June 2013 flood.

Fig. 6 presents the behavior of the TP concentration at the Outflow during the August 2002 and June 2013 floods. In both events the TP concentration at a given discharge was higher during the rising limb of the hydrograph and lower during the falling one. This pattern, well known as clockwise hysteresis effect (Bowes et al., 2005; House and Warwick, 1998; McDiffett et al., 1989), proves the high levels of mobilization and short-term depletion effects that floods exert on the in-stream phosphorus pool. Furthermore, at *Extremely high flows* the mean TP concentration measured in 2013 (0.7 mg L^− 1^) was considerably lower than it was in 2002 (1.4 mg L^− 1^), despite the fact that the maximum daily discharge of 10,570 m^3^ s^− 1^ reached during the 2013 event was much higher than the 10,116 m^3^ s^− 1^ recorded in 2002. This supports the hypothesis that there was an enduring decline in the internal phosphorus stock in the river. This is only a hypothesis, which should be tested through further research. In this respect, the recent flood of 2013 provides an ideal opportunity for further investigations into the river to compare the consequences of the two events.

### 3.2 Annual P loads

#### 3.2.1 Inflow

**Fig. 7:**
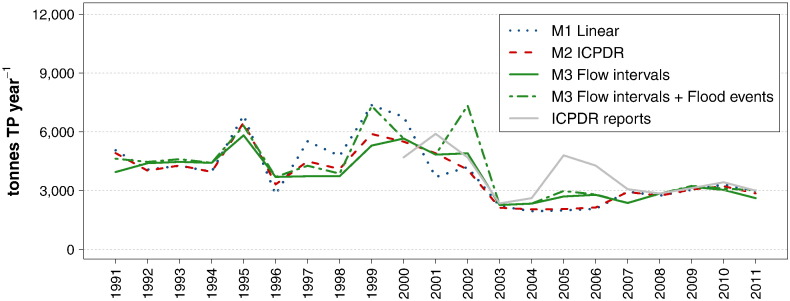
Time series of annual TP loads at the Inflow, obtained with 3 different methods and compared to the calculations published by ICPDR.

If compared to the large variations found by Johnes (2007) and Cassidy and Jordan (2011) in their study of different calculation methods and sampling frequencies, the annual TP loads calculated here present a substantial consistency. This is primarily due to the combination of data sets, with a consequent increase of the sample size, and also to the fact that the Danube is a large river with less pronounced flow dynamics than the smaller streams investigated in the aforementioned studies. Nevertheless, a few notable discrepancies are also detected. As depicted in Fig. 7, the results obtained with methods *M2 — ICPDR* and *M3 — Flow intervals* are highly consistent, whereas *M1 — Linear* presents larger fluctuations within the period T2 (Jan. 1996–Flood 2002). All methods capture the episodic event of 1995 well, but only *M1 — Linear* calculates a spike load in correspondence of the flood of 1999, and none of them delivers a higher load in 2002. This confirms that regular, low frequency monitoring is not a sufficient basis on which to calculate loads in years with important flood events. To obtain an indication of the extent of these underestimations, *M3 — Flow intervals* was also applied with a correction to take into account the high phosphorus concentrations at flood events. The high flow levels were split into *High flows*, *Very high flows*, and *Extremely high flows*, following the same procedure as for the Outflow. The mean concentrations applied for these intervals in each period were obtained by adapting the values calculated for the Outflow, according to the relationship between the mean concentrations at *Mid-range flows* at the two locations. Although this calculation is prone to large uncertainties, its outcomes are highly consistent with all methods for the year 1995 and with *M1 — Linear* in 1999, and in addition they present the expected higher load in 2002

Despite the abovementioned discrepancies, all results depict the same trend, composed of relatively high and fluctuating loads until 2002, followed by a sudden fall in 2003 and by a stable low level thereafter. The calculations published by ICPDR since 2000 show a similar pattern, with two remarkable exceptions in the years 2005 and 2006, for which no plausible explanation could be found.

**Fig. 8:**
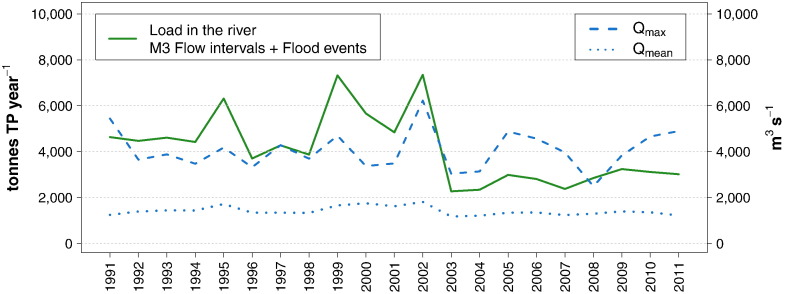
Time series of annual TP river loads, maximum daily discharge and mean daily discharge at the Inflow.

As shown in Fig. 8, until the year 2002, there was a strong correspondence between peaks of annual TP load and peaks of maximum daily discharge. Afterwards maximum discharges as high or even higher than pre-2002 were recorded, but were no longer coupled with high TP loads. The mean annual discharge after 2002 was slightly lower than in the period 1995–2002, but not lower than in the time prior to 1995. It can be concluded that the low level of annual TP loads after 2002 was not caused by an alteration of the hydrological regime, but instead by reduced mobilization of phosphorus in the river. This supports the hypothesis that a depletion of the internal stock occurred as a result of the flood and that its delayed regeneration was a consequence of the reduced discharges of soluble phosphorus.

#### 3.2.2 Outflow

**Fig. 9:**
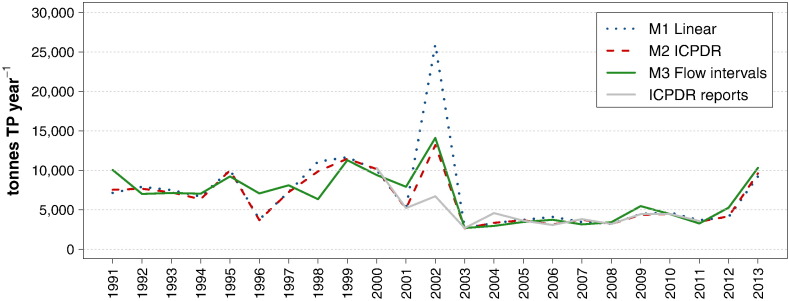
Time series of annual TP loads at the Outflow, obtained with 3 different methods and compared to the calculations published by ICPDR.

As shown in Fig. 9, during the period T2, *M1 — Linear* and *M2 — ICPDR* show more pronounced fluctuations than *M3 — Flow intervals*, because the latter applies an average mean concentration over the whole period, whereas the other methods only consider the concentrations measured in each year. The largest discrepancy affects the load transported in 2002. Whereas *M2 — ICPDR* and *M3 — Flow intervals* lead to the same result, the outcome of *M1 — Linear* is twice as high, because it includes a stratified data set combined with the linear approximation of the algorithm.

The results altogether present the same trend that was observed for the Inflow, namely high and fluctuating loads until 2002, followed by a collapse in 2003 and a long-lasting low level thereafter. The year 2013 presents a renewed high load, expected due to the major flood in June, but still lower than the loads in 1999 and 2002. These results are consistent with the calculations published by ICPDR, with exception of 2002, for which ICPDR estimated a much lower load, because it did not include the data collected during the flood event.

**Fig. 10:**
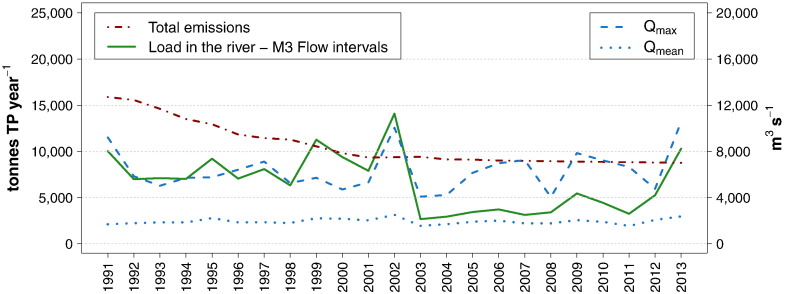
Time series of annual TP river loads, TP emissions, maximum daily discharge and mean daily discharge at the Outflow.

As observed for the Inflow, the patterns of annual riverine TP loads and of maximum discharges were clearly decoupled after 2002 (Fig. 10). In 2009 the two curves show again a simultaneous peak, but whereas the maximum discharge is even higher than the ones recorded before 2002, the TP load is still considerably lower. The same consideration holds true, although to a smaller degree, for the comparison between 2002 and 2013.

In Fig. 10 the developments of riverine TP loads are further compared to total phosphorus emissions from the German and Austrian catchments. These were estimated using the results and background information of Zessner et al. (2011a) as a basis. Their detailed calculation of the emissions for the average period 2001–2006 was adjusted on a yearly scale in consideration of the development of point emissions in Germany (TMLNU, 2009) and Austria (BMLFUW, 1996, 2008, 2012). Therefore, they reflect the decreasing trend of point emissions during the 1990s. However, they fail to capture the fluctuations of diffuse emissions linked to extreme hydrological conditions, in that the average value for the period 2001–2006 was kept constant for the whole time series, except for a 10% increment of the emissions related to erosion processes before the launch of the agri-environmental program ÖPUL in 2000 (Zessner et al., 2011b).

With this limitation in mind, the comparison of the two time series still offers an interesting insight into the mechanisms and drivers regulating the behavior of phosphorus in the river. For the 1990s decade, two observations can be made. Firstly, despite the reduction of point emissions and the decline of TP and SRP baseflow concentrations, there was no decreasing trend of riverine loads. Secondly, the loads were considerably lower than the total input from the catchments, which means that, provided that the discrepancy is not due to an overestimation of the emissions, large amounts of phosphorus were retained every year by the river. In 1999 and 2002, years characterized by flood events, the peaks of TP loads were either equal to or exceeded the emissions. Nonetheless, these peaks did not transport the pool retained during the previous decade downstream, which was instead presumably exported to river banks and flooded areas. After 2002 the retention process resumed its major role, with riverine loads notably lower than total emissions, until 2013, when it was offset once more by the mobilization exerted by a large flood.

This examination highlights and confirms the relevance of the retention process, which creates a large pool of phosphorus distributed among flooded areas, river banks and bed sediments, which is only partially transported downstream during episodic storm events. Moreover, it brings more evidence of the important role played by the mobilizable in-stream phosphorus stock. Its presence during the 1990s hampered the direct translation of the reduction of point discharges and TP and SRP baseflow concentrations into a decline of riverine TP loads. Reversely, after the 2002 flood, the depletion of such reservoir led to long-term low riverine loads, despite the occurrence of moderate floods. This can be further illustrated by a simple calculation. Since point emissions represent the primary pathway of phosphorus at baseflow conditions, it can be expected that their reduction would cause a decline in the loads transported at these flow levels. The average yearly TP point emissions within the period T1 (Jan. 1991–Dec. 1995), T2 (Jan. 1996–Aug. 2002), T3 (Sep. 2002–Dec. 2007), and T4 (Jan. 2008–Dec. 2012) were around 7000, 3100, 2000, and 1700 t TP, respectively. For each of these periods three yearly loads are calculated, assuming a constant mean value of *Low-flows* (950 m^3^ s^− 1^), *Dry conditions* (1300 m^3^ s^− 1^) and *Mid-range flows* (1750 m^3^ s − 1), multiplied by the mean TP concentration at the corresponding flow interval and time period. The mean of these loads is then calculated, obtaining the following average values for the periods T1, T2, T3, and T4: 5000 t, 3500 t, 2300 t, and 2400 t respectively. The comparison of these loads with the point emissions shows an expected decline in both, but not to the same extent, which indicates that considerable retention took place in the period T1, most likely in the form of algae growth. Moreover, both baseflow loads in the periods T1 and T2 are substantially lower than the actual loads calculated including all flow levels; in T3 this difference reaches its minimum, and in T4 they diverge again. This further shows that the mobilization of particulate phosphorus at high flow levels played a major role in determining the total loads during the 1990s, whilst after the 2002 flood its contribution dramatically declined and only slowly started to regain its relevance.

## 4 Conclusions

The first and rather predictable outcome of this study is that the concentration of TP and SRP in the Upper Danube decreased substantially at baseflow conditions during the 1990s, thanks to the efforts undertaken by Germany and Austria to reduce point emissions.

The time series analysis, however, has revealed an even more pronounced and unexpected decline of the TP concentration following the major flood in August 2002. Interestingly, this reduction was very significant even in high flow conditions, and lasted for a long period of time. Such decline was also reflected by the transported TP loads, despite the fact that mean and maximum discharges presented almost no variation before and after the 2002 flood. The validity of these results is strengthened by the fact that the two locations in the study showed very similar and consistent patterns, both in terms of concentrations and loads.

These findings bring new evidence of the significant impact that flood events can exert not only on the episodic transport of riverine loads, but also on the in-stream phosphorus stock and its propensity to mobilization. The existence of a mobilizable pool of phosphorus in the river, built up through the sedimentation of phosphorus rich algae mass, sustained during the 1990s a high level of annual riverine loads, neutralizing the efforts of reduction of point discharges. It was only after the August 2002 flood which removed the richest fraction of this pool, that such efforts were translated into lower long-term loads, in that they slowed down the growth of algae and therefore delayed the regeneration of the stock.

It is thus crucial to better understand and to consider more thoroughly the role that retention, floods and in-stream stocks play in altering the phosphorus mobilization in rivers, either by interfering or amplifying the anthropogenically induced changes. This is highly relevant both for the improvement of river basin models and for the correct interpretation of water quality data in relation to the assessment of environmental management measures.

This study also highlights the usefulness of analyzing shifts in phosphorus concentration as a function of flow level, which offers insight into the different drivers and processes.

Lastly, it flags the value of interrogating long-term and low frequency water quality data sets that, as argued by Burt et al. (2011), should not be dismissed in favor of more recent semi-continuous monitoring, but considered instead as a complementary and valuable source of information to investigate long-term patterns and mechanisms.

## Figures and Tables

**Table 1:** Data sets employed for the Inflow of the Danube into Austria.

Station	Station code	Distance from mouth [km]	Sampling period	Sampling frequency [b]
H_2_O — Jochenstein	FW40607017	2204	Jul. 1991–June 1995	Monthly
H_2_O — Obernzell	FW40607037	2210	Feb. 1996–Dec. 2006	Monthly
ICPDR Bucharest — Jochenstein	D2130	2204	Jan. 1992–Feb. 1998	Monthly
ICPDR TNMN — Jochenstein Austria	AT1	2204	Jan. 1996–Dec. 2011	Monthly (1996–2006) 3 per month (2007–2011)
ICPDR TNMN — Jochenstein Germany	D02	2204	Jan. 1996–Dec. 2011	Bi-weekly

**Table 2:** Data sets employed for the Outflow of the Danube from Austria.

Station	Station code	Distance from mouth [km]	Sampling period	Sampling frequency [b]
H_2_O — Wolfsthal	FW31000027	1874	July 1991–Dec. 2002	Monthly (1991–2000); bi-weekly (2001, 2002)
H_2_O — Deutsch Altenburg	FW31000017	1887	July 1991–May 1995	Monthly
H_2_O — Wildungsmauer	FW31000187	1895	Jan. 1996–Dec. 2012	Monthly
ICPDR Bucharest — Wolfsthal	D1840	1873	Jan. 1992–Feb. 1994	Monthly
ICPDR TNMN — Wolfsthal	AT4	1874	Jan. 1996–Dec. 2005	Monthly (1996–1998); bi-weekly (1999–2005)
ICPDR TNMN — Hainburg	AT6	1879	Jan. 2006–Dec. 2011	Bi-weekly
Zessner et al. (2005)	–	1930	Aug. 2002	2–8 per day
BMLFUW — Wolfsthal	–	1879	Jan. 2013–Dec. 2013	Bi-hourly

**Table 3:** Flow intervals (m^3^ s − 1) for the Inflow and the Outflow.

Location	Low flows	Dry conditions	Mid-range flows	Moist conditions	High flows	Very high flows	Extremely high flows
Inflow	< 800	800–1100	1100–1400	1400–2100	> 2100	–	–
Outflow	< 1100	1100–1600	1600–2000	2000–3000	3000–5500	5500–8000	> 8000

**Table 4:** Time periods and rationale for their categorization.

Period	Time range	Rationale
T1	Jan 1991–Dec 1995	Starting phase of the implementation of the regulation on wastewater phosphorus removal
T2	Jan 1996–Aug 2002	Mature phase of implementation of the regulation on wastewater phosphorus removal; flood in August 2002
T3	Sep 2002–Dec 2007	Short-term effects of 2002 flood
T4	Jan 2008–Dec 2012	Long-term effects of 2002 flood
T5	Jan–Dec 2013	Flood in June 2013; semi-continuous data

**Table 5:** Results of the K-sample Anderson–Darling test applied to the TP data sets available for the Inflow; H_0_: samples are drawn from the same distribution. Statistically significant differences are indicated in bold and sample size in parentheses.

Period	Data set A	Data set B	Dry conditions	Mid-range flows	Moist conditions
T1	H_2_O — Jochenstein	ICPDR Bucharest — Joch.	p > 0.05 (10–7)	p > 0.05 (10–5)	p > 0.05 (12–7)
T2	H_2_O — Obernzell	ICPDR Bucharest — Joch.	p > 0.05 (16–8)	p > 0.05 (21–7)	p > 0.05 (26–5)
	H_2_O — Obernzell	ICPDR TNMN — Joch. A01	p > 0.05 (16–19)	p > 0.05 (21–22)	p > 0.05 (26–20)
	H_2_O — Obernzell	ICPDR TNMN — Joch. D02	p > 0.05 (16–30)	p > 0.05 (21–40)	p > 0.05 (26–52)
	ICPDR Bucharest — Joch.	ICPDR TNMN — Joch. A01	p > 0.05 (8–19)	p > 0.05 (7–22)	p > 0.05 (5–20)
	ICPDR Bucharest — Joch.	ICPDR TNMN — Joch. D02	p > 0.05 (8–30)	p > 0.05 (7–40)	p > 0.05 (5–52)
	ICPDR TNMN — Joch. A01	ICPDR TNMN — Joch. D02	p > 0.05 (19–30)	**p < 0.05** (22–40)	p > 0.05 (20–52)
T3	H_2_O — Obernzell	ICPDR TNMN — Joch. A01	p > 0.05 (13–27)	**p** < **0.001** (8–19)	p > 0.05 (15–24)
	H_2_O — Obernzell	ICPDR TNMN — Joch. D02	p > 0.05 (13–26)	**p < 0.05** (8–5)	**p < 0.01** (15–6)
	ICPDR TNMN — Joch. A01	ICPDR TNMN — Joch. D02	p > 0.05 (27–26)	p > 0.05 (19–5)	p > 0.05 (24–6)
T4	ICPDR TNMN — Joch. A01	ICPDR TNMN — Joch. D02	p > 0.05 (48–47)	p > 0.05 (25–25)	p > 0.05 (37–37)

**Table 6:** Results of the K-sample Anderson–Darling test applied to the TP data sets available for the Outflow; H_0_: samples are drawn from the same distribution. Statistically significant differences are indicated in bold and sample size in parentheses.

Period	Data set A	Data set B	Dry conditions	Mid-range flows	Moist conditions	High flows [b]
T1	ICPDR Bucharest — Wolfsthal	H_2_O — Wolfsthal	**p < 0.01** (10–16)	p > 0.05 (5–6)	p > 0.05 (9–13)	–
	ICPDR Bucharest — Wolfsthal	H_2_O — Deutsch-Altenburg	**p < 0.001** (10–10)	p > 0.05 (5–6)	p > 0.05 (9–11)	–
	H_2_O — Wolfsthal	H_2_O — Deutsch-Altenburg	p > 0.05 (16–10)	p > 0.05 (6–6)	p > 0.05 (13–11)	–
T2	H_2_O — Wolfsthal	ICPDR TNMN — Wolfsthal	p > 0.05 (29–31)	p > 0.05 (33–32)	p > 0.05 (44–46)	p > 0.05 (14–16)
	H_2_O — Wolfsthal	H_2_O — Wildungsmauer	p > 0.05 (29–20)	p > 0.05 (33–23)	p > 0.05 (44–19)	p > 0.05 (14–7)
	ICPDR TNMN — Wolfsthal	H_2_O — Wildungsmauer	p > 0.05 (31–20)	p > 0.05 (32–23)	p > 0.05 (46–19)	p > 0.05 (16–7)
	H_2_O — Wolfsthal	Zessner et al. (2005)	–	–	–	p > 0.05 (14–7)
	ICPDR TNMN — Wolfsthal	Zessner et al. (2005)	–	–	–	p > 0.05 (16–7)
	H_2_O — Wildungsmauer	Zessner et al. (2005)	–	–	–	p > 0.05 (7–7)
T3	H_2_O — Wolfsthal	ICPDR TNMN — Wolfsthal	–	–	p > 0.05 (4–24)	–
	H_2_O — Wolfsthal	H_2_O — Wildungsmauer	–	–	p > 0.05 (4–17)	–
	ICPDR TNMN — Wolfsthal	H_2_O — Wildungsmauer	p > 0.05 (26–17)	**p** < **0.05** (14–13)	p > 0.05 (24–17)	–
	H_2_O — Wolfsthal	ICPDR TNMN — Hainburg	–	–	p > 0.05 (4–14)	–
	ICPDR TNMN — Wolfsthal	ICPDR TNMN — Hainburg	p > 0.05 (26–10)	**p < 0.05** (14–10)	p > 0.05 (24–14)	–
	H_2_O — Wildungsmauer	ICPDR TNMN — Hainburg	p > 0.05 (17–10)	p > 0.05 (13–10)	p > 0.05 (17–14)	–
T4	H_2_O — Wildungsmauer	ICPDR TNMN — Hainburg	p > 0.05 (25–32)	p > 0.05 (13–21)	p > 0.05 (16–25)	–

**Table 7:** Number of paired instantaneous flow and TP, SRP, and SS values for the Inflow, after merging the data sets.

Period	Determinand	Low flows	Dry cond.	Mid-range flows	Moist cond.	High flows
T1	TP	14	17	15	19	5
	SRP	8	14	12	14	4
	SS	14	17	15	19	5
T2	TP	40	73	90	103	50
	SRP	40	73	90	103	50
	SS	40	73	90	103	50
T3	TP	93	66	32	45	10
	SRP	93	66	32	45	10
	SS	72	52	24	39	9
T4	TP	46	95	50	74	34
	SRP	46	95	50	74	34
	SS	22	47	25	37	16

**Table 8:** Number of paired instantaneous flow and TP, SRP, and SS values for the Outflow, after merging the data sets.

Period	Determinand	Low flows	Dry cond.	Mid-range flows	Moist cond.	High flows	Very high flows	Extr. high flows
T1	TP	10	36	17	33	9	0	0
	SRP	7	33	15	29	8	0	0
	SS	8	36	17	33	9	0	0
T2	TP	13	80	88	110	44	20	13
	SRP	13	80	88	110	44	19	13
	SS	13	80	88	110	44	19	13
T3	TP	29	53	39	59	20	3	0
	SRP	29	48	36	55	20	3	0
	SS	29	53	39	59	20	3	0
T4	TP	11	57	34	41	13	0	0
	SRP	10	47	28	35	12	0	0
	SS	11	57	34	41	13	0	0
T5	TP	0	771	1178	1119	601	46	48
	SRP	0	852	1301	1175	603	46	48
	SS	–	–	–	–	–	–	
